# Prevalence and distribution of odontogenic cysts in a Mexican sample. A 753 cases study

**DOI:** 10.4317/jced.53627

**Published:** 2017-04-01

**Authors:** Luis Villasis-Sarmiento, Javier Portilla-Robertson, Arcelia Melendez-Ocampo, Luis-Alberto Gaitan-Cepeda, Elba-Rosa Leyva-Huerta

**Affiliations:** 1 Laboratory of Clinical and Experimental Pathology, Graduate and Research Division, Dental School, National Autonomous University of Mexico, Mexico city, Mexico; 2 Oral Public Health Department, Dental School, National Autonomous University of Mexico, Mexico city, Mexico

## Abstract

**Background:**

Odontogenic cysts (OC) are the most frequent lesions of the jaws and their constant epidemiological update is necessary and indispensable. Therefore the principal objective of this report was To determine prevalence and clinical-demographical characteristics of OC in a Mexican sample.

**Material and Methods:**

753 cases of OC coming from the archive of a head and neck histopathological teaching service, from January 2000 to December 2013, were included. OC cases were re-assessed according 2005 WHO classification. Chi square test was used to establish possible associations (*p*<0.05IC95%).

**Results:**

From 753 OC, 369 were female and 384 male; 52.9% of them were in their 2nd- 4th decade of life. The most common location (41%) was the mandibular posterior area. Radicular cysts were more frequent in maxillary anterior zone of females (*p* 0.0002) at their fourth decade of life. Dentigerous cysts were more frequent in the mandibular posterior zone of males (*p* 0.0000) in their second decade of life. Six cases of periodontal lateral cyst; 4 cases of paradental cysts; 4 eruption cysts and 4 cases of adult gingival cyst, as well were identified.

**Conclusions:**

Radicular cyst and dentigerous cyst are the most prevalent odontogenic cyst in this Mexican sample. Due to their etiology, dental pulpar necrosis and impacted teeth, radicular cyst and dentigerous cyst could be prevenible. Therefore, it is necessary to establish preventive strategies to diminish dental decay and programs of prophylactic extractions of impacted teeth, to in consequence decrease the prevalence of odontogenic cysts.

** Key words:**Cyst, dentigerous cyst, mexican, odontogenic cyst, radicular cyst.

## Introduction

Odontogenic cysts (OC) are pathological cavities, lined with odontogenic epithelium, which appear in both jaws and sporadically in the oral soft tissues principally the gums (1). Odontogenic cysts can have their onset at any age, and remain asymptomatic and therefore undetected for long periods of time. In most cases, routine x-rays reveal suspicion of OC presence. Their genesis is closely related to dental ontogeny, 90% of them are formed from odontogenic epithelium or its embryonic remnants, nevertheless, in most cases, their etiology is still unknown (2-5). Their treatment, surgical exceresis, is invasive and even mutilating; it might affect oral and dental functionality (2).

In 2005, World Health Organization (WHO) reclassified odontogenic keratocyst as a tumor and renamed it keratocystic odontogenic tumor, while calcifying odontogenic cyst was also classified as a tumor and was renamed Calcifying Cystic Odontogenic Tumor (1). In spite of the fact that these classifications caused changes in frequency and prevalence of odontogenic cyts and tumors (6), thus rendering epidemiological updates necessary, the specific information about demographical and clinical dataof OC using WHO 2005 classification is scarce. Furthermore, there are few studies available, independently of classification used, assaying the occurrence OC in different Latin American populations (7-10), specifically in Mexicans (11,12). For the aforementioned reasons, a 14-year retrospective study was conducted at a maxillofacial and oral histopathological diagnosis teaching service with the main objective of to establish OC prevalence and to assess their demographical and clinical characteristics.

## Material and Methods

The archive of Histopathological Diagnosis Service, at the Graduate and Research Division, Dental School, National Autonomous University of Mexico were reviewed in order to identify and select all cases with OC diagnosis. Time span covered was January 2000 - December 2013. The following demographical and clinical data were obtained from medical files: gender, age at moment of diagnosis, and lesion location. In this latter variable, both jaws (upper and lower) were divided into anterior zone and posterior zone. For research purposes, in the present article, anterior zone was considered from right upper canine to left upper canine in case of maxilla and from right lower canine to left lower canine in case of mandible; while posterior zone was considered the area comprised from the first bicuspid to the third molar, irrespective of whether it was right or left.

In order to be included in the present study, cases should have sufficient biological material embedded in paraffin and/or histological slides stained with hematoxylin and eosin. In those cases which had enough biological material embedded but lacked histological slides, new cuts were obtained in order to be stained with the same staining technique. Two oral pathology experts (JPR/LAGC) reviewed all selected cases in order to confirm or refute diagnosis. Cases were grouped according to 2005-classification to Odontogenic Cyst and Tumors proposed by the WHO (1). Cases which caused diagnosis conflict were discarded from the study.

The present project adhered to research parameters established by the Mexican General Health Law, article 17, fraction I (14). This research was considered of low risk to physical and psychological integrity of the patient, since only documental research techniques and methods were used, and the protocol research was approved by the Bioethical Comittee of the Dental School, National Autonomous University of Mexico, and it was conducted in full accordance with the World Medical Association Declaration of Helsinski. It was a retrospective study performed on lesional tissue embedded and preserved wax block, so consent was not needed from the patients who donated the samples. Additionaly all cases were codified to be anonymised and de-identified prior to analysis.

An ex professo database was built with obtained data. Frequencies and relative frequencies of each OC type were obtained using the software program, SPSS® version 20 (IBM®). A chi square test was used to ascertain possible associations (*p* < 0.05, CI95%).

## Results

In the allotted time 10,970 cases were reviewed, out of which 753 were diagnosed as OC. In such a way that OC represented 6.8% of all diagnoses. Out of the 753 OC cases, 369 (49.1%) were found in females and 384 (50.9%) in males (*p*>0.05) ( Table 1). With respect to age distribuition, majory of OC, 148 cases (19.6%), were found in the 10-19 year old age group, followed by the 30-39 year old age group with 129 (17.1%) cases; 52.9% of all OC were found in patients from 2nd to 4th decade of life. Total case distribution according to age is shown in  Table 2.

**Table 1:**
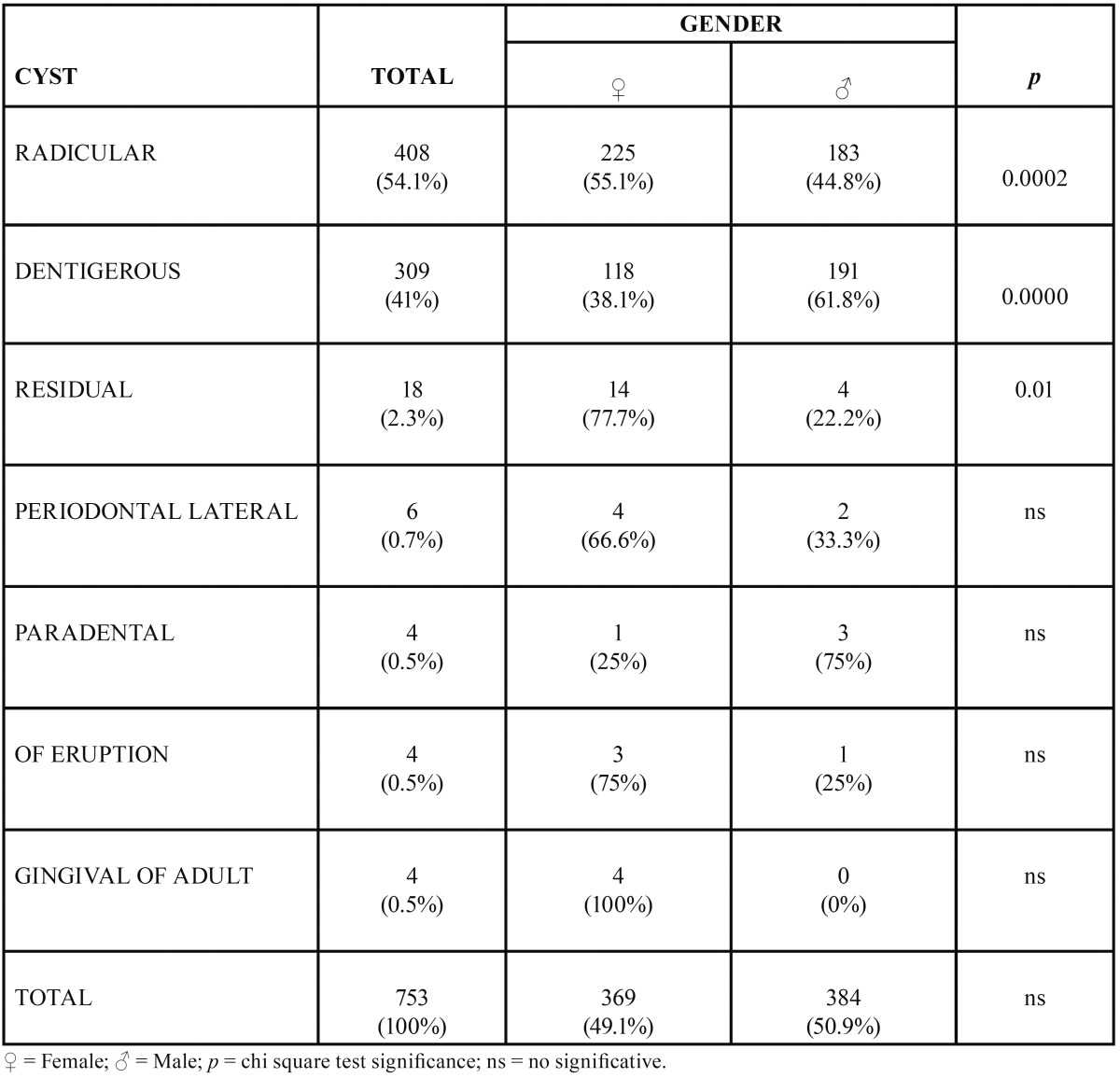
Relative frequency and distribution by gender of odontogenic cyst in a Mexican sample.

**Table 2:**
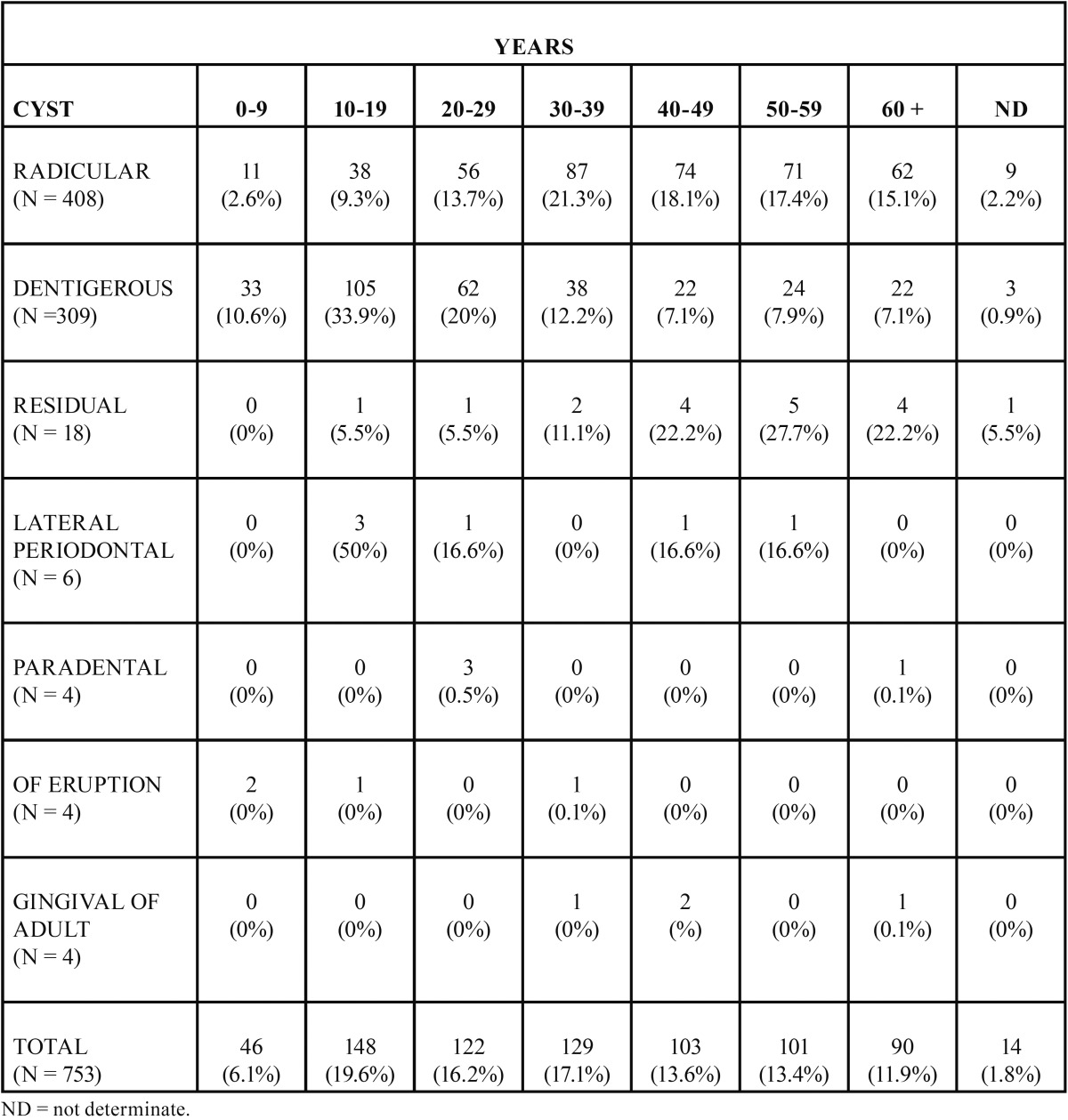
Age distribution at moment of diagnosis of patients suffering odontogenic cyst in a Mexican sample.

The most prevalent anatomical location was the mandibular posterior area with 260 cases (34.5%), followed by the maxillary anterior zone with 193 cases (25.6%) and the maxillary posterior area with 144 cases (19.1%) ( Table 3).

**Table 3:**
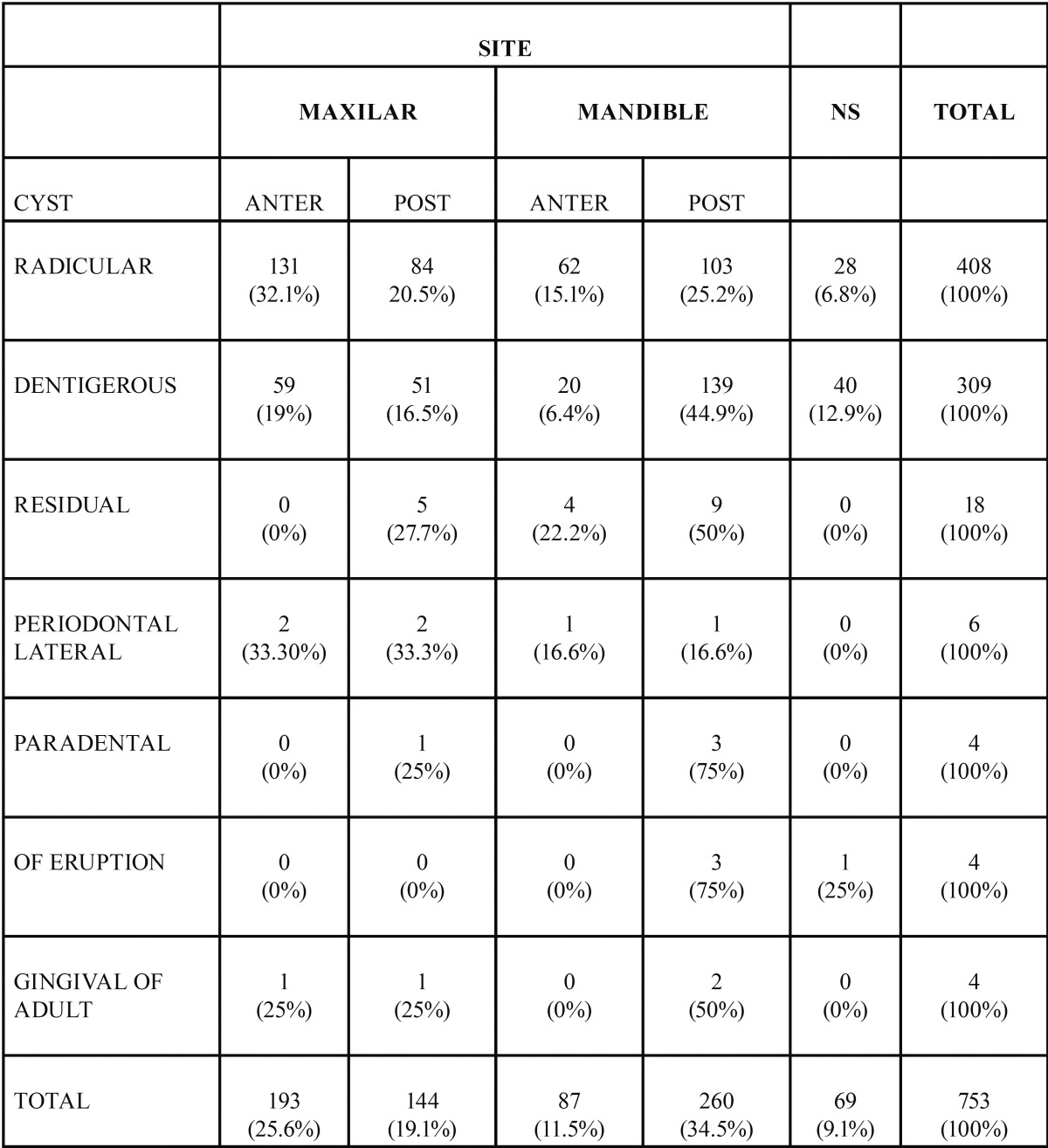
Topographical distribution of odontogenic cyst in a Mexican sample.

With respect to histological type, radicular cyst was the most common type with 408 cases, followed by dentigerous cyst with 309 cases, exhibiting a prevalence of 3.7% and 2.8% with respect to the total number of cases (n = 10,970). Total and relative frequency of all diagnosed cysts is shown in  Table 1.

Radicular cysts represented 54.1% of all reported cysts and it was more frequent in females with an incidence peak at the fourth decade of life. Association of radicular cyst and female gender was statistically significant (*p* 0.0002). The most frequent location of radicular cyst was the maxillary anterior zone mainly associated to central and lateral incisor teeth. On the other hand, 309 cases of dentigerous cyst were reported, which represented 41% of all OC. Dentigerous cyst was more frequent in the mandibular posterior zone of males at their second decade of life, mainly associated to third molars. The association of dentigerous cyst and male gender was stastistically significant (*p* 0.0000). With respect to residual cysts, 18 cases were detected, most frequently found infemales at their 6th decade of life, located primarily in the posterior area of the mandible, mainly associated to premolar area.

Six cases of periodontal lateral cyst were reported. Four were found in females, 3 cases were reported in subjects at their 2nd decade of life with no anatomical or tooth predilection. Four cases of paradental cysts were identified, 3 males and one female; 3 out of 4 cases corresponded to patients in their third decade of life, with most frequent location in the posterior mandibular area, in all cases associated to third molars. Four eruption cysts were identified, in three females and in a one male, 2 out of the 4 eruption cyst cases were found in patients in their first decade of life, 3 of them in a posterior mandibular location. With respect to the adult gingival cyst, 4 cases were diagnosed, all of them in females, two of them in their fifth decade of life, and 2 of the 4 cysts were in the mandibular posterior zone. Distribution of all OC with respect to gender is shown in  Table 1;  Table 2 shows age at moment of diagnosis while the topography of OC is shows in  Table 3.

## Discussion

This paper targets the updating of relative frequency and clinical and demographical characteristics of OC in a Mexican sample. The 2005 WHO odontogenic tumors and cyst classification impacts in the prevalence and distribution of odontogenic cyst and tumours (6), therefore, our results were compared with others studies performed using 2005 WHO classification to achieve better accuracy. Surprinsingly, the amount of studies with the aforementioned characteristics were few (8,14-17).

In the present study the patients in the second to 4th decade of life were the most frequently afflicted by OC, slightly younger than the published on Nigerians (18) and Australians (14) (3th - 5th decade of life). No statistically significant association between OC and gender was observed, however slightly higher prevalence in males had been reported (14). In this Mexican sample the most frequent anatomical location of OC was the mandibular posterior area with a ratio mandible:maxilla of 1.2:1, these data are very similar to those reported in Nigerians (18) of 1.5:1; and to the 1.3:1 ratio reported in Australians (14).

The relative frequency of radicular cyst in this Mexican sample (54.1%) is slightly higher than those reported in scientific literature, specifically when it is compared to those reports achieved using 1997 WHO classification, e.g.: 52.3% in the United Kingdom (19); 50.7% in Chile (7); 53.5% in France (20), 52.2% in Brazil (8) as well as 50.2% in Spain (21). Three studies reported higher prevalence than our: 59% in Turkey (22); 72.5% and 61.4% in Brazil (9,10). The values reported in series using 2005 WHO classification ranged from 38.7% in Kenyans (16) to 64.3% in Saudis (17). A putative explanation to justify this high prevalence of radicular cyst could be related to caries. Radicular cyst in most cases is a consequence of deep carious lesions and dental pulp necrosis. In our country (México) wide population sectors shows higher prevalence of deep dental caries and pulp necrosis is as a very common consequence (24). The mechanism to develop radicular cyst includes pulp necrosis, colonization and proliferation of microorganisms within the root canal system, release of bacteria toxins and inflammatory mediators into the periapical region and a combination of factors involving epithelial-stromal interaction. The periradicular inflammation leads to proliferation of epithelial cell rests (Malasses rest) (25). In most cases, endodontic treatment is the first option to resolve apical inflammatory lesions. However, due to financial reasons this treatment is not available to some sectors of the main population in our country.

With respect to dentigerous cysts we observed the same pattern. We obtained one of the highest dentigerous cyst prevalence (41%), compared to data reported in studies using 1997 WHO classification, including reports on Mexicans conducted by Ledesma (12) in 2000 (35.5%) and Mosqueda (11) in 2002 (38.8%). In the United Kingdom a prevalence of 18.1% was reported (19); in Chile 18.5% (9); in Brazil 20.1% (10); in France 22.3% (20) and in Turkey 14% (22). On the other hand when dentigerous cyst prevalence in Mexicans is compared to 2005 WHO classification studies, it represents the 2nd higher prevalence ( Table 4). High prevalence is necesarelly related to high prevalence of impacted teeth due to the fact impacted teeth is necesary condition to develop a dentigerous cyst. An average of world wide rate of third molar impactation of 24.4% (26) has been reported.

**Table 4:**
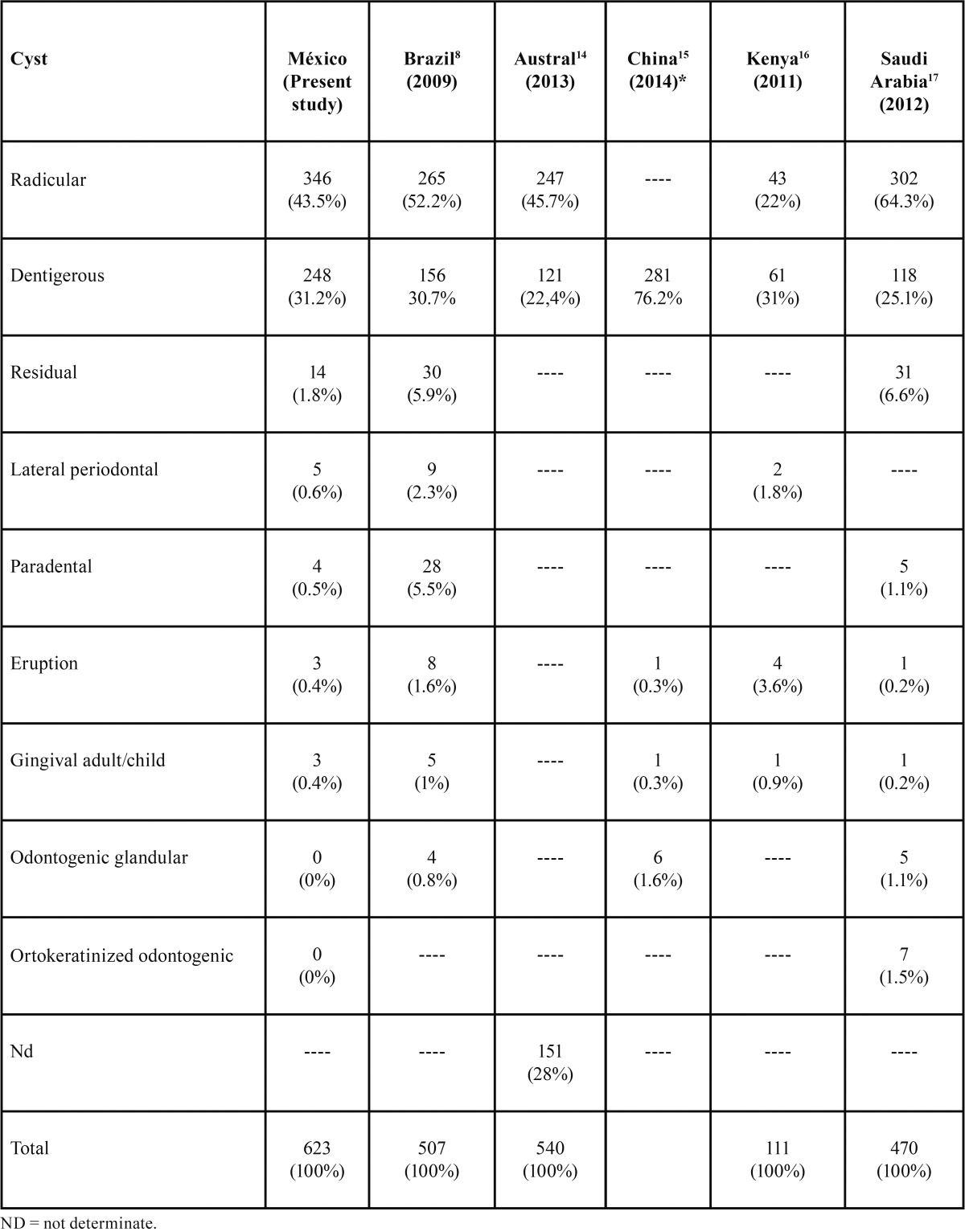
Comparison of relative frequency of odontogenic cysts in a Mexican sample versus selected studies performed using 2005-WHO classification.

Four cases of paradental cyst were identified. The paradental cyst or inflammatory paradental cyst is defined as “a cyst occurring near to the cervical margin of the lateral aspect of a root as a consequence of an inflammatory process in the periodontal pocket; 61.4% of them are associated to partially or fully erupted 3rd vital mandibular molars (27); 3 from 4 paradental cyst cases included in the present report were located in mandible. Data on incidence and prevalence of paradental cyst are scarce and generally circunscribed to isolated case reports, however the reported relative frequency varies form 0.9 to 4.7% (for a review see 27). In our series the relative frequency of paradental cyst was 0.5%. In paradental cysts a male preponderance has been suggested (27). In our serie 3 out of 4 paradental cyst cases were in males.

On the other hand lateral periodontal cyst (LPC) is an uncommon development OC, representing about 0.4% of all OC. Lateral periodontal cysts develop in the alveolar bone along the lateral surface of a vital and erupted tooth. We obtainded a relative frequency for periodontal lateral cysts of 0.7%. LPC are most common in males into their 5th to 7th decades of life (mean age of 50.8 years) (28). However our data disagreed with the aforementioned since 4 out of 6 LPC cases were found in women in their 2nd decade of life. Most of LPC are located in the mandibular-premolar area, followed by the anterior region of maxilla (28). We did not observe association to any particular site. With respect to gingival cysts and eruption cysts, their rarity precludes posibility to achieve any asumption of inference with respect to demographical characteristics. Comparison of our results with reported by others research groups are show in  Table 4.

To our knowledge there are two previous reports about OC prevalence in Mexicans (11,12), both of them published before 2005. In comparison to the aforementioned reports our data shows an increase of 39.4% in radicular cyst prevalence, from 38.8% in 2000 (12) to 54.1%; and of 24.2% in dentigerous cyst prevalence, from 35.5% to 41% (12). To date we lack explanations to justifiy this apparent variation. Nevertheless this increase could be an indirect result of the 2005 WHO classification, since in both series (11,12) the odontogenic keratocyst ranked third in prevalence. We did not include in our series odontogenic keratocysts, consequently the values of most prevalent odontogenic cysts, radicular and dentigerous cyst were increased. Nevertheless acci-dental events cannot be excluded. A research protocol specifically designed to clarify this could be necesary. In the meantime, conclusions about changes in OC prevalence in Mexicans should be interpreted with the utmost caution.

The aforementioned aquires importance due to the fact that National Health Systems require accurate information regarding disease ocurrence in orden to effectively allocate resources and frame goverment health policies. Misleading or confusing data may inadvertently provide the wrong information to policy makers. Demographical data sustained by statistical data, are the basis for evidence-based policy. Accurate data will eventually enhance population health (14).

## Conclusions

OC are they most frequent intraosseous lesions of the jaws and therefore their demographical and clinical characteristics should be acknowledged by the general dentist. On the other hand, due to the fact of close relationship between dental caries and radicular cyst it is necesary to establish preventive strategies to diminish dental decay and in consequence decrease radicular cyst prevalence. This proposal becomes important since a preventive program not only impinges on their primary objective, but it additionally exerts a direct effect on bone lesions necessarily requiring surgical treatment which in some cases will elicit mutilations.
